# Correction: Shen et al. Platelet Activation and Cytokine Release of Interleukin-8 and Interferon-Gamma-Induced Protein 10 after ChAdOx1 nCoV-19 Coronavirus Vaccine Injection. *Vaccines* 2023, *11*, 456

**DOI:** 10.3390/vaccines11121775

**Published:** 2023-11-28

**Authors:** Chih-Lung Shen, Tso-Fu Wang, Chao-Zong Liu, Yi-Feng Wu

**Affiliations:** 1 Department of Hematology and Oncology, Hualien Tzu Chi Hospital, Buddhist Tzu Chi Medical Foundation, Hualien 970, Taiwan; 2 College of Medicine, Tzu Chi University, Hualien 970, Taiwan; 3 Department of Pharmacology, School of Medicine, Tzu Chi University, Hualien 970, Taiwan; 4 Ph.D. Program in Pharmacology and Toxicology, Tzu Chi University, Hualien 970, Taiwan

The authors wish to make the following corrections to this published paper [1].

Due to the authors’ oversight, Figures 3 and 4 were misplaced and need to be switched. The corrected Figure 3 and Figure 4 are shown below:

In addition, the authors would also like to correct the typing errors. In the Abstract, “interluekin-8” should be “interleukin-8”. In the fifth paragraph of the Discussion section, “COVD-19” should be “COVID-19”.

The authors state that the scientific conclusions are unaffected. This correction was approved by the Academic Editor. The original publication has also been updated.

## Figures and Tables

**Figure 3:**
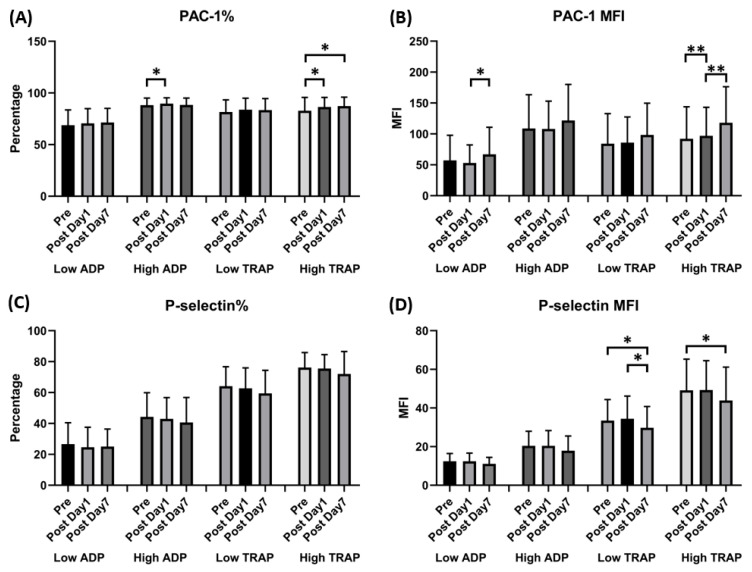
Expression of GPIIb-IIIa (PAC-1) and *p*-selectin in human platelets following ChAdOx1 nCoV-19 vaccination. (**A**) The percentage of platelet surface activated GPIIb-IIIa. (**B**) Platelet surface activated GPIIb-IIIa mean fluorescence intensity (MFI). (**C**) The percentage of platelet surface *p*-selectin. (**D**) Platelet surface *p*-selectin MFI (in the presence of the indicated concentrations of ADP or thrombin receptor-activating peptide [TRAP]). * *p* < 0.05; ** *p* < 0.01.

**Figure 4:**
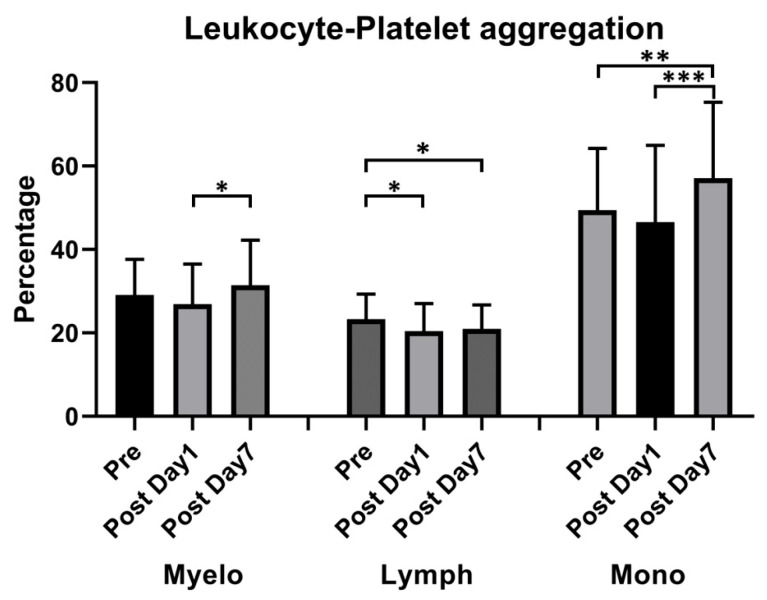
Effect of ChAdOx1 nCoV-19 vaccination on platelet-promoted leukocyte aggregation. Quantitative analysis of platelet–leukocyte aggregates: Myeloid (Myelo), Lymphocyte (Lymph), and Monocyte (mono). * *p* < 0.05; ** *p* < 0.01, *** *p* < 0.001.
